# Fatigue and Cognitive Impairment in Patients with Kidney Failure Not Requiring Kidney Replacement Therapy and Patients Receiving Hemodialysis: A Cross-Sectional Study

**DOI:** 10.3390/brainsci16070760

**Published:** 2026-07-20

**Authors:** Piotr Olejnik, Gabriela Drzewiec, Jadwiga Rojek-Trębicka, Paweł Żebrowski, Jolanta Małyszko, Aleksandra Golenia

**Affiliations:** 1 Department of Neurology, Medical University of Warsaw, 02-097 Warsaw, Polandagolenia@gmail.com (A.G.); 2 Doctoral School, Medical University of Warsaw, 02-093 Warsaw, Poland; 3 Department of Nephrology, Dialysis and Internal Medicine, Medical University of Warsaw, 02-097 Warsaw, Polandjolmal@poczta.onet.pl (J.M.)

**Keywords:** cognitive impairment, fatigue, kidney failure, chronic kidney disease, hemodialysis, Montreal Cognitive Assessment, Fatigue Assessment Scale

## Abstract

**Highlights:**

**What are the main findings?**

- Cognitive impairment and fatigue are frequent in both patients with kidney failure not requiring kidney replacement therapy and hemodialysis patients.
- Fatigue is not associated with global cognitive performance, although exploratory analyses suggested a relationship between mental fatigue and language performance.

**What are the implications of the main findings?**

- Cognitive screening may be clinically relevant in patients with advanced CKD, including those with kidney failure not requiring kidney replacement therapy.
- Fatigue should not be considered a direct proxy for objective cognitive impairment in patients with kidney failure.

**Abstract:**

**Background:** Cognitive impairment and fatigue are common in patients with advanced chronic kidney disease (CKD). However, whether these symptoms reflect kidney failure itself or factors associated with hemodialysis (HD) remains unclear. This study compared cognitive performance and fatigue between patients with CKD G5 not requiring kidney replacement therapy, referred to as CKD G5 not on dialysis (CKD G5 ND), and those receiving maintenance HD. **Methods:** In this cross-sectional, single-center study, 68 clinically stable patients with kidney failure were enrolled, including 27 patients with CKD G5 ND and 41 patients receiving HD. Cognitive performance was assessed using the Montreal Cognitive Assessment (MoCA) and fatigue via the Fatigue Assessment Scale (FAS). **Results:** Global cognitive performance did not differ significantly between patients receiving HD and those with CKD G5 ND, with median MoCA scores of 24.00 and 23.00, respectively. Cognitive impairment was frequent, affecting 61.0% of HD patients and 74.1% of those with CKD G5 ND. Fatigue severity was also comparably high, with significant fatigue in 51.2% and 44.4% of individuals, respectively. No association was observed between global cognitive performance and fatigue. Exploratory analyses showed that language performance was inversely associated with total and mental fatigue after false discovery rate correction. In the adjusted logistic regression, HD status was associated with lower odds of cognitive impairment; however, this finding should be interpreted cautiously, given the cross-sectional design, small sample size, and baseline clinical differences between groups. **Conclusions:** Cognitive impairment and fatigue were highly prevalent both in patients with CKD G5 ND and those receiving maintenance HD, with no significant between-group differences in global cognitive performance or fatigue severity scores. These findings suggest that neuropsychological vulnerability may be present in patients with kidney failure before dialysis initiation.

## 1. Introduction

Kidney failure represents the most advanced stage of chronic kidney disease (CKD), corresponding to CKD G5. It is defined by a glomerular filtration rate (eGFR) below 15 mL/min/1.73 m^2^ and encompasses both individuals not requiring kidney replacement therapy (KRT), referred to as CKD G5 not on dialysis (CKD G5 ND), and those requiring KRT, including maintenance hemodialysis (HD) [1,2]. Beyond renal manifestations, kidney failure is considered a systemic condition with well-established consequences for brain health [3]. The concept of kidney-brain crosstalk emphasizes that impaired kidney function may affect the central nervous system through shared vascular, inflammatory, and metabolic pathways, increasing the vulnerability to neurological and neuropsychological dysfunction [4,5].

In this context, cognitive impairment has become a serious and increasingly recognized complication of CKD [6,7]. Evidence indicates that it may occur across the entire CKD spectrum and become more pronounced as kidney function declines. Brodski et al. (2019) systematically reported that cognitive performance broadly deteriorates from CKD G1 to G5, with patients with kidney failure showing deficits across multiple domains, including attention, processing speed, executive function, memory, visuospatial abilities, cognitive control, and global cognition [8]. In patients with CKD G5 approaching KRT, cognitive performance is particularly relevant because deficits in attention, executive functioning, memory, and processing speed may compromise comprehension of medical information, shared decision-making about dialysis initiation and modality, vascular access preparation, treatment adherence, self-management, and quality of life [6,9].

Of the 3.5 million people worldwide receiving maintenance dialysis, approximately 90% undergo HD [10]. On the one hand, HD mitigates metabolic consequences of kidney failure by removing fluid and solutes. Nonetheless, the dialysis procedure may cause cerebral stress. Repeated intradialytic hemodynamic instability, rapid osmotic shifts, and circulatory stress may provoke transient reductions in cerebral perfusion and consequently contribute to ischemic brain injury, white matter damage, hippocampal vulnerability, and cognitive impairment [11]. Therefore, comparing patients with CKD G5 ND with those treated with HD may help distinguish cognitive vulnerability related to kidney failure *per se* from the effects associated with HD exposure.

In addition to cognitive impairment, fatigue is another prevalent and debilitating symptom of CKD and kidney failure, affecting up to 70% of patients [12,13]. Fatigue is a multidimensional neurobehavioral symptom involving physical, cognitive, emotional, and motivational factors rather than a mere nephrological or quality-of-life complaint. It overlaps with mental fatigue, reduced cognitive effort, attentional inefficiency, depression, and sleep disruption [12]. Fatigue also contributes to reduced activity, avoidance of daily tasks, and sedentary behavior [14]. This is clinically relevant because our previous study identified sedentary lifestyle as a modifiable factor associated with cognitive impairment in dialysis and kidney transplant patients [15]. Thus, fatigue may be linked to cognitive functioning not only directly through mental fatigue or reduced cognitive effort but also indirectly by promoting physical inactivity and sedentary behavior, which are important modifiable risk factors for dementia [16].

Despite evidence that cognitive impairment and fatigue are common in advanced CKD, the relative contributions of kidney failure and dialysis-related factors to neuropsychological dysfunction remain unclear. Most previous studies have focused on cognitive impairment in dialysis populations, whereas comparatively few have directly compared patients with CKD G5 ND with those undergoing HD.

Therefore, this study aimed to compare global and domain-specific cognitive performance and fatigue severity between patients with CKD G5 ND and those receiving maintenance HD. We also examined whether fatigue was associated with global cognitive performance and specific cognitive domains in the overall cohort of patients with kidney failure. By comparing patients with CKD G5 not requiring KRT with those already receiving HD, this study sought to determine whether cognitive impairment and fatigue were already evident in the CKD G5 ND stage and generate hypotheses regarding the potential contribution of dialysis status to neuropsychological vulnerability in kidney failure.

## 2. Materials and Methods

In this cross-sectional study, consecutive patients with CKD G5 ND and patients receiving HD who agreed to participate in the study were recruited from a single nephrology outpatient clinic and a single dialysis unit, respectively, between February 2026 and June 2026.

The inclusion criteria were kidney failure, age ≥ 18 years, and clinical stability confirmed by the nephrology team. The exclusion criteria included infectious disease symptoms within 8 weeks, decompensated heart or liver failure, alcohol abuse, delirium, severe psychiatric disorders, or inability to complete assessments due to language barriers, sensory impairment, limb paresis, or documented cognitive decline or dementia before screening.

Cognitive function and fatigue were screened in all participants. Cognitive performance was assessed using the Montreal Cognitive Assessment (MoCA) and fatigue was evaluated using the Fatigue Assessment Scale (FAS). In HD patients, assessments were performed on the day of the dialysis session within the first hour of treatment. The assessment of individuals with CKD G5 ND was conducted during their scheduled outpatient visit in a separate room that provided a quiet, distraction-free environment.

Demographic characteristics, including age, sex, and years of education, as well as clinical data, including comorbidities, dialysis duration in HD patients, and laboratory parameters, were collected from medical records or directly from patients.

The study was conducted as an anonymous questionnaire-based and screening assessment and did not constitute a medical experiment within the meaning of Article 21, paragraph 1 of the Polish Act of 5 December 1996 on the professions of physicians and dentists (Journal of Laws of 2018, item 617). In accordance with the requirements for anonymous questionnaire-based studies that do not impose a substantial burden on participants, the local Bioethics Committee acknowledged the study protocol (acknowledgment no. AKBE/40/2026). Therefore, formal ethical approval was not required for this study.

Participants were informed about the study’s purpose, data anonymity, and voluntary participation. Informed consent to participate in the study was obtained from all participants, and completion of the assessments was considered equivalent to consent for the use of anonymized data for scientific purposes.

### 2.1. Cognitive Function Screening

Although no specific screening battery is dedicated to CKD-related cognitive impairment, the MoCA has been validated in patients undergoing HD [17]. The MoCA is a 30-point cognitive screening tool assessing visuospatial abilities (using figure copying and clock drawing tasks), executive functions (using the Trail Making Test, phonemic fluency, and verbal abstraction), language (through animal naming and sentence repetition), orientation (to date, month, year, day of the week, place, and city), attention (using tapping, serial subtractions, and forward/backward digit span), as well as memory (using delayed recall) [18]. It is widely used to detect mild cognitive impairment. This one-page, paper-and-pencil battery takes less than 10 min. Importantly, a validated Polish-language version of this tool is available. A cutoff score of ≤24 points was used as an indication of cognitive impairment, according to a Polish study by Magierska and colleagues [19].

Cognitive function was assessed using the MoCA version 8.2 by one of two certified researchers (P.O., certification number: PLOLEPI710649134-02, 10 September 2025; G.D., certification number: PLDRZGA710861878-01, 16 September 2025).

### 2.2. Fatigue Screening

Among the several tools available for assessing fatigue in clinical settings, the FAS was used in this study because it has previously been applied to individuals with kidney failure treated with HD [20]. The FAS is a 10-item self-report questionnaire that assesses overall fatigue severity. It is quick to administer, requiring 2–3 min. Each item uses a five-point Likert scale (1–5), with reverse scoring for items 4 and 10. The total score ranges from 10 to 50. A cut-off of ≥22 indicates relevant fatigue. The FAS captures physical and mental fatigue, allowing separate analysis of these dimensions alongside overall fatigue tendency [20].

### 2.3. Charlson Comorbidity Index

The Charlson Comorbidity Index (CCI) was used to assess the comorbidity burden. The CCI is a well-established tool for predicting long-term mortality. It assigns weighted scores to comorbid conditions; higher values indicate greater multimorbidity and increased risk of adverse outcomes [21].

In this study, the CCI served as a global indicator of multimorbidity and clinical complexity. This is relevant because, beyond traditional cardiovascular risk factors and CKD-related complications, the CCI includes conditions like chronic pulmonary disease, connective tissue disease, ulcer disease, and liver disease [21]. Thus, the CCI captures a broader cumulative disease burden.

### 2.4. Statistical Analysis

Statistical analyses were performed using IBM SPSS Statistics version 31.0 (IBM Corp., Armonk, NY, USA). The distribution of continuous variables was assessed using the Shapiro-Wilk test. Normally distributed continuous variables are presented as mean ± standard deviation (SD), whereas non-normally distributed continuous variables are presented as median and interquartile range [Q1; Q3]. Exceptionally, cognitive and fatigue scale results were presented using both mean ± SD and median [Q1; Q3] to facilitate clinical interpretation. Categorical variables are presented as counts and percentages. Figures were prepared using the GraphPad Prism software version 8.0 (GraphPad Software, Boston, MA, USA). Between-group comparisons of continuous variables were performed using Student’s *t*-test for normally distributed data and the Mann-Whitney U test for non-normally distributed data. Categorical variables were compared using the χ^2^ test of independence or Fisher’s exact test as appropriate. Correlations between global cognitive performance and fatigue measures were assessed using Spearman’s rank correlation coefficients. To account for multiple testing, *p*-values were adjusted using the Benjamini-Hochberg false discovery rate (FDR) procedure. Binary logistic regression was performed to assess the association between dialysis status and cognitive impairment. Cognitive decline was entered as the dependent variable and treated as a dichotomous outcome, defined as the presence or absence of cognitive impairment. Results are presented as odds ratios (ORs) or adjusted odds ratios (aORs), with corresponding 95% confidence intervals (CIs) and *p*-values. Four regression models were constructed. Model 1 examined the unadjusted association between HD and cognitive impairment. Model 2 was adjusted for age and years of education, which were selected *a priori* as key demographic determinants of cognitive performance. Model 3 was additionally adjusted for comorbidity burden, which was assessed using the CCI. Model 4 included clinically significant fatigue as a binary covariate, defined as the FAS score ≥22. HD status was entered as the primary independent variable, comparing patients receiving HD with those not on dialysis. Continuous covariates, including age and years of education, were modeled per one-unit increase. A two-sided *p*-value < 0.05 was considered statistically significant.

## 3. Results

### 3.1. General Characteristics

The study included 68 patients, of whom 27 and 41 were CKD G5 ND and undergoing HD, respectively. The demographic and clinical characteristics of the participants are summarized in Table 1. The HD and CKD G5 ND cohorts did not differ significantly with respect to age (65.8 ± 15.1 vs. 62.8 ± 18.9; *p* = 0.467) or years of education (13 vs. 14; *p* = 0.131). The proportion of female individuals was significantly lower in the dialysis cohort than in the CKD G5 ND cohort (22.0% vs. 44.4%; *p* = 0.049). Among the patients undergoing HD, the median dialysis vintage was 27 months.

Patients undergoing dialysis had a higher comorbidity burden, as reflected by a significantly higher CCI (6 vs. 5; *p* = 0.002). Additionally, diabetes mellitus, atrial fibrillation, ischemic heart disease, and smoking were significantly more prevalent in the dialysis cohort. However, hypertension, dyslipidemia, and history of stroke or transient ischemic attack did not differ significantly between the groups. Among the biochemical parameters, the study groups differed significantly in terms of serum creatinine concentration (7.9 vs. 5 mg/dL; *p* = 0.001), eGFR (6 vs. 11 mL/min/1.73 m^2^; *p* = 0.001), and uric acid concentration (5.3 vs. 6.3 mg/dL; *p* = 0.004). No statistically significant differences were observed in urea, hemoglobin, or fasting glucose concentrations.

### 3.2. Global Cognitive Performance and Fatigue

Global cognitive performance assessed using the MoCA did not differ significantly between the dialysis and CKD G5 ND cohorts (Table 2). The mean MoCA total score was 23.41 ± 3.81 in HD patients and 23.44 ± 3.91 in CKD G5 ND patients (*p* = 0.860). The prevalence of cognitive impairment was lower in the dialysis cohort than in the CKD G5 ND group (61.0% vs. 74.1%, respectively). However, this difference was not statistically significant (*p* = 0.264).

Fatigue severity was comparable between the groups. The mean total FAS scores were 23.02 ± 8.86 and 22.00 ± 8.03 in the dialysis and CKD G5 ND cohorts, respectively (*p* = 0.630). Neither the physical nor the mental FAS subscales differed significantly between the cohorts. Clinically significant fatigue was present in 51.2% of the patients undergoing dialysis and 44.4% of the patients with CKD G5 ND (*p* = 0.584).

### 3.3. Domain-Specific Cognitive Performance

**Figure 1:**
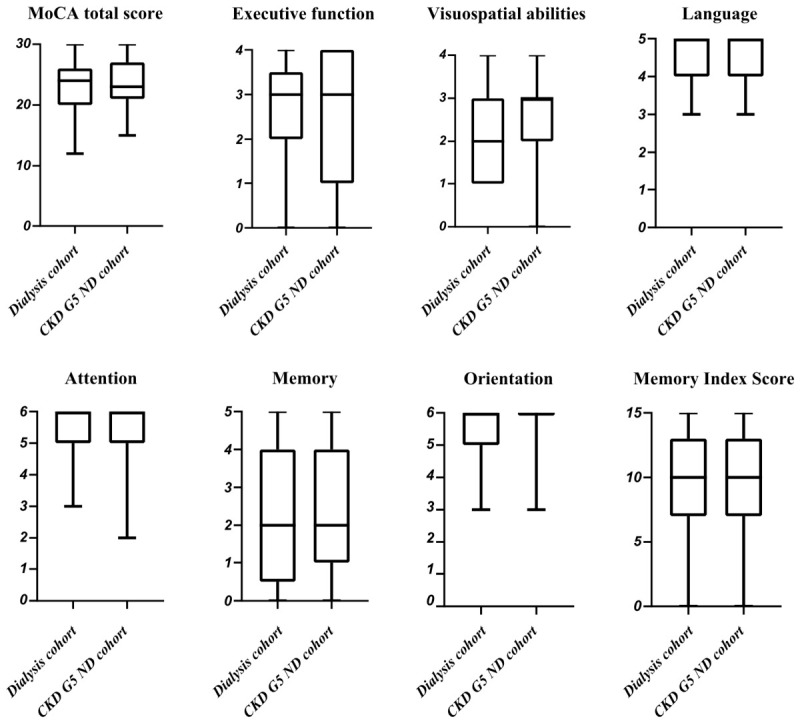
Global and domain-specific cognitive performance assessed with the Montreal Cognitive Assessment in patients receiving hemodialysis and patients with chronic kidney disease G5 not requiring kidney replacement therapy (CKD G5 ND). The results are presented as box plots, where the horizontal line denotes the median, the box represents the interquartile range (from the first to the third quartile), and the whiskers extend to the minimum and maximum values.

Domain-specific MoCA subscores showed no statistically significant differences between the dialysis and CKD G5 ND cohorts (Figure 1). Scores for executive function, visuospatial abilities, language, attention, memory, Memory Index Score (MIS), and orientation were broadly comparable between the groups. The median scores were identical or closely similar across most domains, including executive function, attention, memory, and MIS.

### 3.4. Association Between Cognitive Performance and Fatigue

Correlation analysis revealed no significant association between global cognitive performance and fatigue (Table 3). The MoCA total score was not correlated with the FAS total score (Spearman’s ρ = −0.024; *p* = 0.845; FDR-adjusted *p* = 0.948). Similarly, no significant correlations were observed between the MoCA total score and either the physical or mental fatigue subscales.

In exploratory domain-specific analyses, most MoCA domains were not significantly associated with fatigue measures after correction for multiple testing (Table 4). The only domain showing a statistically significant inverse association after FDR adjustment was language performance in relation to mental fatigue (ρ = −0.445; *p* < 0.001; FDR-adjusted *p* = 0.021). Language performance was also inversely correlated with total fatigue before and after FDR adjustment (ρ = −0.350; *p* = 0.003; FDR-adjusted *p* = 0.032). The association between language performance and physical fatigue was not statistically significant (ρ = −0.229; *p* = 0.061; FDR-adjusted *p* = 0.427).

### 3.5. Logistic Regression Models for Cognitive Impairment

In unadjusted logistic regression, dialysis status was not significantly associated with cognitive impairment (OR: 0.547; 95% CI: 0.188–1.587; *p* = 0.267). After adjusting for age and years of education (Model 2), patients undergoing HD had lower odds of cognitive impairment (aOR: 0.189; 95% CI: 0.043–0.840; *p* = 0.029). In this model, older age was associated with higher odds of cognitive impairment (aOR: 1.069 per year; 95% CI: 1.025–1.116; *p* = 0.002), whereas longer education was associated with lower odds (aOR: 0.720 per year; 95% CI: 0.564–0.920; *p* = 0.008) (Table 5).

After additional adjustment for comorbidity burden using the CCI, the association between HD and lower odds of cognitive impairment remained statistically significant (*p* = 0.011). Education was also independently associated with lower odds of cognitive impairment (*p* = 0.010). Nonetheless, age and CCI were not statistically significant in this model.

In the fully adjusted model, additionally including clinically significant fatigue, HD remained independently associated with lower odds of cognitive impairment (*p* = 0.011), whereas education remained protective (*p* = 0.010). Clinically significant fatigue was not associated with cognitive impairment (aOR: 0.940; 95% CI: 0.254–3.486; *p* = 0.927).

## 4. Discussion

In this cross-sectional study, cognitive impairment and fatigue were present to a comparable extent in individuals with CKD G5 ND and those receiving HD. The prevalence of cognitive impairment was high in both the dialysis and CKD G5 ND groups, reaching 61.0% and 74.1%, respectively. The median MoCA scores were 23.00 for individuals with CKD G5 ND and 24.00 for HD patients. This result is consistent with a cross-sectional study by Nicholas et al. (2022) [22], in which patients with CKD G5 not receiving KRT demonstrated poorer cognitive performance than patients undergoing conventional HD (with median MoCA scores of 23.00 vs. 24.50). Notably, in that study, the authors also evaluated 33 patients receiving home HD who scored significantly higher (median MoCA score of 28.00) than both CKD G5 and HD groups. The superior performance of the home HD group may partly reflect selection effects, as these individuals were younger, had fewer comorbidities, and had completed more years of education [22]. Nonetheless, collectively, these results imply that neuropsychological vulnerability in patients with kidney failure might be present before dialysis initiation and should not be solely attributed to HD.

The prevalence of significant fatigue in HD patients was similar to that in previous HD cohort reports, which ranged between 42% and 89% based on the population and assessment tools used [23]. Notably, the comparable fatigue severity in CKD G5 ND and HD patients suggests that fatigue might represent the systemic impact of advanced kidney disease rather than just the dialysis process [12].

It is noteworthy that the CKD G5 ND group had a considerably higher number of female individuals. This is because, worldwide, male patients have a higher incidence of KRT, implying a quicker progression to kidney failure [24]. However, our earlier published research found no notable sex-related cognitive differences among those who received kidney transplants [25].

The adjusted association between HD status and reduced cognitive impairment odds should be approached with caution and not considered direct evidence of the protective effect of dialysis. Contrary to our finding, longitudinal data from Kurella Tamura et al. (2017) [26] indicated that starting dialysis was linked to a notable decline in executive function, but no significant changes in global cognition or memory were observed. Importantly, their cognitive assessments were conducted on non-dialysis days, whereas ours were performed during HD sessions [26]. Therefore, the discrepancy might be due to differences in the study design, assessment timing, and sample size. Notably, patients undergoing HD reveal cognitive fluctuations [27,28], with some patients showing deterioration after dialysis in attention and executive functions [29].

The independent association between higher education levels and reduced odds of cognitive impairment aligns with the idea that educational attainment might contribute to cognitive reserve or affect performance on cognitive screening tests. Respectively, recent meta-analysis by Pei et al. (2025) revealed that lower education level is strongly associated with cognitive impairment (OR: 2.59; 95% CI: 1.32–5.09) among patients with CKD not requiring KRT [30].

While HD has been proposed to influence cognitive impairment through mechanisms such as intradialytic cerebral hypoperfusion, circulatory stress, and alterations in cerebral oxygenation [11], our cross-sectional findings did not demonstrate inferior global or domain-specific MoCA scores in HD patients when compared to those with CKD G5 ND. This suggests that although dialysis-related cerebral stress cannot be dismissed, dialysis exposure alone may not entirely account for cognitive impairment in kidney failure.

Fatigue and cognitive impairment are often found together in CKD and kidney failure. However, the lack of a link between FAS and overall MoCA scores in this study implies that fatigue should not be viewed as a proxy for objective cognitive impairment. In exploratory analyses focusing on specific domains, language performance was inversely related to total fatigue and more significantly to mental fatigue. The link between mental fatigue and reduced language performance should be considered as a basis for forming hypotheses rather than as definitive proof. This suggests that short language tasks may be influenced by mental exertion, attentional regulation, and processing effectiveness. This is especially true since the repetition of complex sentences demands focus and concentration, both of which are impacted by fatigue [31]. Mental fatigue diminishes goal-oriented attention and causes behavior to become more reactive to stimuli [32]. Future research employing comprehensive neuropsychological assessments, including measures of verbal fluency, naming, sentence repetition, semantic access, and processing speed, is necessary to ascertain whether mental fatigue selectively impacts language-related cognitive performance in patients with kidney failure.

## 5. Limitations

This study has several limitations. First, its cross-sectional nature prevents the establishment of causality and does not distinguish between cognitive impairment due to advanced kidney disease, existing vulnerabilities, or the long-term effects of HD exposure. Second, the research involved a small group from a single nephrology outpatient clinic and dialysis unit, which limits statistical power, raises the risk of residual confounding, and reduces the generalizability of the study. Third, there were differences between the dialysis and CKD G5 ND groups in terms of sex distribution, comorbidity levels, diabetes prevalence, cardiovascular disease, smoking habits, and kidney function parameters. Although the regression models included covariates, unmeasured confounders might have affected the findings. Additionally, cognitive function was evaluated using the MoCA screening tool instead of a comprehensive neuropsychological battery, which limits precision at the domain level and prevents the diagnosis of neurocognitive disorders. Similarly, fatigue was measured using a self-report questionnaire, which could be influenced by numerous factors and affected by recall bias. Finally, in HD patients, assessments were performed during the first hour of the dialysis session, complicating the task of fully distinguishing the intradialytic impacts on cognition and fatigue from the consistent differences between groups. The study by Murray et al. (2007) suggests that the optimal timing for cognitive evaluation is during an interdialytic period (24 to 30 h post-dialysis), although cognitive function remains intact approximately one hour before HD as well [28]. Since our study involved screening patients at the start of the HD session, with the screening process taking up to 15 min, the intradialytic effects should not be overly emphasized.

## 6. Conclusions

These findings suggest that in advanced kidney disease, cognitive impairment and fatigue may emerge before dialysis initiation rather than being primarily attributable to HD exposure itself. The absence of a global association between fatigue and cognitive performance indicates that fatigue should not be treated as a direct proxy for objective cognitive impairment in this population group. Overall, neuropsychological vulnerability in patients with kidney failure appears to reflect a complex interaction between disease burden, cognitive reserve, and individual clinical factors, supporting the need for cognitive screening also in patients with kidney failure not requiring KRT. Longitudinal studies using comprehensive neuropsychological assessments are needed to clarify the relative contributions of advanced kidney disease and dialysis-related factors to cognitive outcomes.

## Figures and Tables

**Table 1:** Demographic, clinical, and biochemical characteristics of the study groups.

Variable	Hemodialysis Cohort (*n* = 41)	CKD G5 ND Cohort (*n* = 27)	*p*-Value
General characteristics			
Female sex, *n* (%)	9 (22.0)	12 (44.4)	0.049 ^a,^*
Age, years, mean ± SD	65.8 ± 15.1	62.8 ± 18.9	0.467 ^b^
Years of education, years, median [Q1; Q3]	13 [12; 15]	14 [12; 17]	0.131 ^c^
Dialysis vintage, months, median [Q1; Q3]	27 [13.5; 62]	n/a	n/a
Charlson Comorbidity Index, median [Q1; Q3]	6 [5; 8]	5 [3; 6]	0.002 ^c,^*
Comorbid conditions			
Hypertension, *n* (%)	38 (92.7)	25 (92.6)	1.000 ^d^
Diabetes mellitus, *n* (%)	24 (58.5)	7 (25.9)	0.008 ^a,^*
Dyslipidemia, *n* (%)	20 (48.8)	16 (59.3)	0.397 ^a^
Atrial fibrillation, *n* (%)	10 (24.4)	1 (3.7)	0.040 ^d,^*
Ischemic heart disease, *n* (%)	18 (43.9)	4 (14.8)	0.012 ^a,^*
Previous stroke/TIA, *n* (%)	3 (7.3)	0 (0.0)	0.271 ^d^
Smoking, *n* (%)	24 (58.5)	9 (33.3)	0.042 ^a,^*
Biochemical data			
Creatinine level (mg/dL), median [Q1; Q3]	7.9 [5.5; 9.7]	5 [3.9; 5.6]	0.001 ^c,^*
eGFR (mL/min/1.73 m^2^), median [Q1; Q3]	6 [5; 9.5]	11 [10; 13]	0.001 ^c,^*
Urea level (mg/dL), mean ± SD	124.4 ± 51.3	145.0 ± 46.7	0.099 ^b^
Uric acid (mg/dL), median [Q1; Q3]	5.3 [4.75; 6.15]	6.3 [5; 8.1]	0.004 ^c,^*
Hemoglobin (g/dL), median [Q1; Q3]	10.3 [9.9; 11.1]	10.4 [9.5; 11.5]	0.995 ^c^
Fasting glucose level (mg/dL), median [Q1; Q3]	109 [88; 133]	97 [93; 114]	0.310 ^c^

CKD G5 ND: chronic kidney disease G5 not on dialysis; CCI: Charlson Comorbidity Index; eGFR: estimated glomerular filtration rate; *n*: number of patients; n/a: not applicable; TIA: transient ischemic attack; SD: standard deviation; Q1: first quartile; Q3: third quartile; ^a^ χ^2^ test; ^b^ Student’s *t*-test; ^c^ Mann-Whitney U test; ^d^ Fisher’s Exact Test; * *p*-value < 0.05.

**Table 2:** Global cognitive performance and fatigue in hemodialysis and pre-dialysis CKD G5 patients.

Variable	Hemodialysis Cohort (*n* = 41)	CKD G5 ND Cohort (*n* = 27)	*p*-Value
Global cognitive performance—Montreal Cognitive Assessment results			
MoCA total score/30 mean ± SD median [Q1; Q3]	23.41 ± 3.81 24.00 [20.00; 26.00]	23.44 ± 3.91 23.00 [21.00; 27.00]	0.860 ^a^
Cognitive impairment, *n* (%) *	25 (61.0)	20 (74.1)	0.264 ^b^
Fatigue—Fatigue Assessment Scale results			
FAS total score, mean ± SD median [Q1; Q3]	23.02 ± 8.86 22.00 [17.00; 29.00]	22.00 ± 8.03 20.00 [17.00; 27.00]	0.630 ^c^
Physical FAS score, mean ± SD median [Q1; Q3]	12.32 ± 4.97 12.00 [8.00; 16.00]	12.48 ± 4.56 11.00 [9.00; 16.00]	0.975 ^a^
Mental FAS score, mean ± SD median [Q1; Q3]	10.71 ± 4.47 10.00 [7.00; 13.00]	9.56 ± 3.77 9.00 [6.00; 11.00]	0.374 ^a^
Clinically significant fatigue, *n* (%) **	21 (51.2)	12 (44.4)	0.584 ^b^

CKD G5 ND: chronic kidney disease G5 not on dialysis; MoCA: Montreal Cognitive Assessment; FAS: Fatigue Assessment Scale; *n*: number of patients; SD: standard deviation; Q1: first quartile; Q3: third quartile; * cognitive impairment was defined as MoCA ≤24 points; ** clinically significant fatigue was defined as FAS ≥22 points; ^a^ Mann-Whitney U test; ^b^ χ^2^ test; ^c^ Student’s *t*-test.

**Table 3:** Correlations between global cognitive performance and fatigue in the overall study cohort.

Variable Pair	Spearman’s ρ	*p*-Value	FDR-Adjusted *p*-Value
MoCA total score vs. FAS total score	−0.024	0.845	0.948
MoCA total score vs. FAS physical fatigue subscale	−0.008	0.948	0.948
MoCA total score vs. FAS mental fatigue subscale	−0.060	0.629	0.948

MoCA: Montreal Cognitive Assessment; FAS: Fatigue Assessment Scale; FDR: false discovery rate.

**Table 4:** Exploratory correlations between MoCA cognitive domains and fatigue measures in the overall study cohort.

Cognitive Domain	Fatigue Assessment	Spearman’s ρ	*p*-Value	FDR-Adjusted *p*-Value
MIS	FAS total	0.041	0.738	0.961
MIS	FAS mental	0.057	0.644	0.961
MIS	FAS physical	0.020	0.874	0.981
Executive function	FAS total	0.050	0.685	0.961
Executive function	FAS mental	0.055	0.655	0.961
Executive function	FAS physical	0.037	0.763	0.961
Visuospatial function	FAS total	−0.047	0.706	0.961
Visuospatial function	FAS mental	−0.107	0.387	0.961
Visuospatial function	FAS physical	−0.035	0.778	0.961
**Language**	**FAS total**	**−0.350**	**0.003**	0.032 *
**Language**	**FAS mental**	**−0.445**	**<0.001**	0.021 *
Language	FAS physical	−0.229	0.061	0.427
Attention	FAS total	−0.102	0.406	0.961
Attention	FAS mental	−0.164	0.182	0.956
Attention	FAS physical	−0.063	0.608	0.961
Memory	FAS total	0.051	0.679	0.961
Memory	FAS mental	0.070	0.568	0.961
Memory	FAS physical	0.040	0.749	0.961
Orientation	FAS total	−0.008	0.949	0.981
Orientation	FAS mental	−0.003	0.978	0.981
Orientation	FAS physical	0.003	0.981	0.981

MoCA: Montreal Cognitive Assessment; MIS: Memory Index Score; FAS: Fatigue Assessment Scale; FDR: false discovery rate; * FDR-adjusted *p* < 0.05.

**Table 5:** Logistic regression models for predicting the presence of cognitive impairment.

	Model 1			Model 2			Model 3			Model 4		
Predictor	OR	95% CI	*p*	aOR	95% CI	*p*	aOR	95% CI	*p*	aOR	95% CI	*p*
Hemodialysis vs. non-dialysis	0.547	0.188–1.587	0.267	0.189	0.043–0.840	0.029 *	0.102	0.018–0.588	0.011 *	0.103	0.018–0.600	0.011 *
Age, per 1 year	-	-	-	1.069	1.025–1.116	0.002 *	1.039	0.984–1.097	0.165	1.038	0.983–1.097	0.181
Education, per 1 year	-	-	-	0.720	0.564–0.920	0.008 *	0.716	0.556–0.923	0.010 *	0.717	0.556–0.924	0.010 *
Charlson Comorbidity Index	-	-	-	-	-	-	1.421	0.922–2.190	0.112	1.427	0.916–2.222	0.116
Fatigue	-	-	-	-	-	-	-	-	-	0.940	0.254–3.486	0.927

OR: odds ratio; aOR: adjusted odds ratio; CI: confidence interval; CCI: Charlson Comorbidity Index; FAS: Fatigue Assessment Scale; * *p* < 0.05. Model 1 assessed the unadjusted association between hemodialysis status and cognitive impairment. Model 2 was adjusted for age and years of education. Model 3 was additionally adjusted for comorbidity burden, as measured by the CCI. Model 4 was further adjusted for clinically significant fatigue, entered as a binary covariate and defined as the FAS score ≥22.

## Data Availability

The data presented in this study are available upon request from the corresponding author due to ethical and privacy restrictions.

## Abbreviations

The following abbreviations are used in this manuscript:

aOR	adjusted odds ratio
CCI	Charlson Comorbidity Index
CI	confidence interval
CKD	chronic kidney disease
eGFR	estimated glomerular filtration rate
FAS	Fatigue Assessment Scale
FDR	false discovery rate
HD	hemodialysis
KRT	kidney replacement therapy
MIS	Memory Index Score
MoCA	Montreal Cognitive Assessment
ND	not on dialysis
OR	odds ratio
Q1	first quartile
Q3	third quartile
SD	standard deviation
TIA	transient ischemic attack

## References

[1] Pethő Á.G., Tapolyai M., Csongrádi É., Orosz P. (2024). Management of Chronic Kidney Disease: The Current Novel and Forgotten Therapies. J. Clin. Transl. Endocrinol..

[2] Levey A.S., Eckardt K.-U., Dorman N.M., Christiansen S.L., Cheung M., Jadoul M., Winkelmayer W.C. (2020). Nomenclature for Kidney Function and Disease—Executive Summary and Glossary from a Kidney Disease: Improving Global Outcomes (KDIGO) Consensus Conference. Eur. Heart J..

[3] Pépin M., Levassort H., Massy Z.A. (2024). The Impact of Chronic Kidney Disease on Cognitive Function. Curr. Opin. Nephrol. Hypertens..

[4] Xie Z., Tong S., Chu X., Feng T., Geng M. (2022). Chronic Kidney Disease and Cognitive Impairment: The Kidney-Brain Axis. Kidney Dis..

[5] Yan Q., Liu M., Xie Y., Lin Y., Fu P., Pu Y., Wang B. (2024). Kidney-Brain Axis in the Pathogenesis of Cognitive Impairment. Neurobiol. Dis..

[6] Bolignano D., Simeoni M., Hafez G., Pepin M., Gallo A., Altieri M., Liabeuf S., Giannakou K., De A., Capasso G. (2025). Cognitive Impairment in CKD Patients: A Guidance Document by the CONNECT Network. Clin. Kidney J..

[7] Pépin M., Klimkowicz-Mrowiec A., Godefroy O., Delgado P., Carriazo S., Ferreira A.C., Golenia A., Malyszko J., Grodzicki T., Giannakou K. (2023). Cognitive Disorders in Patients with Chronic Kidney Disease: Approaches to Prevention and Treatment. Eur. J. Neurol..

[8] Brodski J., Rossell S.L., Castle D.J., Tan E.J. (2019). A Systematic Review of Cognitive Impairments Associated With Kidney Failure in Adults Before Natural Age-Related Changes. J. Int. Neuropsychol. Soc..

[9] Pépin M., Giannakou K., Levassort H., Farinha A., Bobot M., Lo Re V., Golenia A., Małyszko J., Mattace-Raso F., Klimkowcz-Mrowiec A. (2025). Care Pathways for Patients with Cognitive Impairment and Chronic Kidney Disease. Nephrol. Dial. Transplant..

[10] Flythe J.E., Watnick S. (2024). Dialysis for Chronic Kidney Failure. JAMA.

[11] Wolfgram D.F. (2019). Intradialytic Cerebral Hypoperfusion as Mechanism for Cognitive Impairment in Patients on Hemodialysis. J. Am. Soc. Nephrol..

[12] Gregg L.P., Bossola M., Ostrosky-Frid M., Hedayati S.S. (2021). Fatigue in CKD. Clin. J. Am. Soc. Nephrol..

[13] Fletcher B.R., Damery S., Aiyegbusi O.L., Anderson N., Calvert M., Cockwell P., Ferguson J., Horton M., Paap M.C.S., Sidey-Gibbons C. (2022). Symptom Burden and Health-Related Quality of Life in Chronic Kidney Disease: A Global Systematic Review and Meta-Analysis. PLoS Med..

[14] Capasso G., Franssen C.F.M., Perna A.F., Massy Z.A., Menzies R.I., Zoccali C., Tessitore A., Nedergaard M., Okusa M.D., Ortiz A. (2025). Drivers and Mechanisms of Cognitive Decline in Chronic Kidney Disease. Nat. Rev. Nephrol..

[15] Golenia A., Olejnik P., Maciejewska O., Wojtaszek E., Żebrowski P., Małyszko J. (2024). Sedentary Lifestyle Is a Modifiable Risk Factor for Cognitive Impairment in Patients on Dialysis and after Kidney Transplantation. J. Clin. Med..

[16] Livingston G., Huntley J., Liu K.Y., Costafreda S.G., Selbæk G., Alladi S., Ames D., Banerjee S., Burns A., Brayne C. (2024). Dementia Prevention, Intervention, and Care: 2024 Report of the Lancet Standing Commission. Lancet.

[17] Malyszko J., Golenia A., Farisco M., Re V.L., Klimkowicz-Mrowiec A., Capasso G., Goumenos D., Rroji M., Figurek A., Hafez G. (2025). Cognitive Impairment in Kidney Transplanted Patients. Nephrol. Dial. Transplant..

[18] Gelow J.M., Mudd J.O., Chien C.V., Lee C.S. (2015). Usefulness of Cognitive Dysfunction in Heart Failure to Predict Cardiovascular Risk at 180 Days. Am. J. Cardiol..

[19] Magierska J., Magierski R., Fendler W., Kłoszewska I., Sobów T.M. (2012). Clinical Application of the Polish Adaptation of the Montreal Cognitive Assessment (MoCA) Test in Screening for Cognitive Impairment. Neurol. Neurochir. Pol..

[20] Zyga S., Alikari V., Sachlas A., Fradelos E., Stathoulis J., Panoutsopoulos G., Georgopoulou M., Theophilou P., Lavdaniti M. (2015). Assessment of Fatigue in End Stage Renal Disease Patients Undergoing Hemodialysis: Prevalence and Associated Factors. Med. Arch..

[21] Charlson M.E., Carrozzino D., Guidi J., Patierno C. (2022). Charlson Comorbidity Index: A Critical Review of Clinimetric Properties. Psychother. Psychosom..

[22] Nicholas P., Green T., Purtell L., Bonner A. (2022). A Cross-sectional Study Exploring Cognitive Impairment in Kidney Failure. J. Ren. Care.

[23] Artom M., Moss-Morris R., Caskey F., Chilcot J. (2014). Fatigue in Advanced Kidney Disease. Kidney Int..

[24] Bikbov B., Purcell C.A., Levey A.S., Smith M., Abdoli A., Abebe M., Adebayo O.M., Afarideh M., Agarwal S.K., Agudelo-Botero M. (2020). Global, Regional, and National Burden of Chronic Kidney Disease, 1990–2017: A Systematic Analysis for the Global Burden of Disease Study 2017. Lancet.

[25] Olejnik P., Golenia A., Maciejewska O., Kurzawa D., Wojtaszek E., Małyszko J. (2025). Sex-Related Disparities in Cognitive Impairment in Kidney Transplant Patients with Kidney Failure. J. Nephrol..

[26] Kurella Tamura M., Vittinghoff E., Hsu C., Tam K., Seliger S.L., Sozio S., Fischer M., Chen J., Lustigova E., Strauss L. (2017). Loss of Executive Function after Dialysis Initiation in Adults with Chronic Kidney Disease. Kidney Int..

[27] Iyasere O., Brown E.A. (2017). Cognitive Function before and after Dialysis Initiation in Adults with Chronic Kidney Disease—A New Perspective on an Old Problem?. Kidney Int..

[28] Murray A.M., Pederson S.L., Tupper D.E., Hochhalter A.K., Miller W.A., Li Q., Zaun D., Collins A.J., Kane R., Foley R.N. (2007). Acute Variation in Cognitive Function in Hemodialysis Patients: A Cohort Study With Repeated Measures. Am. J. Kidney Dis..

[29] Costa A.S., Tiffin-Richards F.E., Holschbach B., Frank R.D., Vassiliadou A., Krüger T., Eitner F., Gross T., Shah N.J., Schulz J.B. (2014). Clinical Predictors of Individual Cognitive Fluctuations in Patients Undergoing Hemodialysis. Am. J. Kidney Dis..

[30] Pei X., Bakerally N.B., Wang Z., Bo Y., Ma Y., Yong Z., Zhu S., Gao F., Bei Z., Zhao W. (2025). Kidney Function and Cognitive Impairment: A Systematic Review and Meta-Analysis. Ren. Fail..

[31] Julayanont P., Nasreddine Z.S. (2017). Montreal Cognitive Assessment (MoCA): Concept and Clinical Review. Cognitive Screening Instruments.

[32] Boksem M.A.S., Meijman T.F., Lorist M.M. (2005). Effects of Mental Fatigue on Attention: An ERP Study. Cogn. Brain Res..

